# Left fourth and sixth costovertebral dislocation abutting the aorta

**DOI:** 10.1007/s00256-023-04415-3

**Published:** 2023-07-31

**Authors:** Natalia Gorelik, Dany Croteau, Valérie Gorelik, Joseph Casullo

**Affiliations:** 1 Department of Diagnostic Radiology, McGill University Health Center, 1001 Decarie Blvd, Quebec H4A 3J1 Montreal, Canada; 2 Faculty of Medicine, McGill University, 3605 de la Montagne Street, Montreal, Quebec H3G 2M1 Canada; 3 Dawson College, 3040 Sherbrooke St W, Montreal, Quebec H3Z 1A4 Canada

**Keywords:** Aorta, Costovertebral joint, Dislocation, Spine, Trauma

## Abstract

While rib fractures are common in blunt thoracic trauma, dislocations of the costovertebral joints (CVJs) are extremely rare and typically involve the first, eleventh, or twelfth rib. We report a rare case of dislocation of the left fourth and sixth CVJs in a 36-year-old man who was run over by a car. The rib heads were displaced anteriorly, abutting the aorta. Additional injuries included bilateral hemopneumothoraces, pneumomediastinum, pulmonary contusions, grade 3 splenic injury, left adrenal hematoma, retroperitoneal hematoma, Morel-Lavallée lesions at bilateral hips, and multiple fractures, including at the ribs and pelvis. There was also a fracture of the fourth thoracic vertebral body, which was occult on initial CT, but seen on subsequent CTs. The CVJ dislocations were managed conservatively, without short-term complications. Prompt surgical intervention has been recommended in cases where sharp rib fracture fragments are displaced close to the aorta to prevent fatal aortic injuries. However, there is a literature gap on the management of rib heads that are dislocated against the aorta. Our experience suggests that conservative management may be acceptable in some of these cases. This case report aims to increase radiologists’ awareness of CVJ injuries, which are important for thoracic spine stability, and highlights the association between CVJ dislocations and spinal injuries.

## Introduction

While rib fractures are common in blunt thoracic trauma, affecting 20–39% of patients [1], costovertebral joint (CVJ) dislocations are very rare [2, 3] and typically involve the first rib [3–11] or the floating (i.e., eleventh and twelfth) ribs [9, 12–15]. The English-language literature on traumatic CVJ dislocations consists of case reports [2–5, 7–14, 16–18] and case series [6, 15, 19]. CVJ dislocations have also been described in neurofibromatosis type 1 where dystrophic scoliosis (marked by a short segment sharply angulated curvature and vertebral body wedging, rotation, and scalloping), together with foraminal enlargement, spindling of the transverse processes, and apical rib penciling contribute to rib head displacement into the spinal canal along the convex aspect of the curve, predisposing to cord compression [20]. Other etiologies of CVJ injuries include chronic post-traumatic scoliosis [21], child abuse [22], surgical procedures [23], and sports such as rowing and butterfly swimming [24].

Both rib fractures [1] and CVJ dislocations [2, 6–8, 10–14, 17, 18] are usually managed conservatively. Indications for surgical stabilization of rib fractures include multiple rib fractures with bicortical displacement, flail chest, displaced rib fractured with sternal and/or ipsilateral displaced midshaft clavicular fracture, pain, chest wall deformity particularly if thoracic volume is reduced, acute respiratory distress syndrome in patients with thoracic cage instability, and fractured ribs acting as penetrating objects [1]. There are multiple reported cases of aortic injury caused by rib fracture fragments presenting with hemorrhage and hemodynamic instability hours or days after the initial trauma [25–36]. Early surgical intervention to prevent this life-threatening complication has therefore been suggested when rib fracture fragments are displaced close to the aorta [30, 34, 35, 37]. Vascular injury has been reported in CVJ dislocation without fracture [4]. Although indications for surgical treatment of CVJ dislocations remain to be determined, surgery has been used in cases of persistent pain and neurological symptoms [9, 38]. We present a rare case of left fourth and sixth CVJ dislocation, where the displaced rib heads were abutting the aorta, which was managed conservatively. To the best of our knowledge, such a pattern of dislocation of non-consecutive CVJs at those levels and the management of displaced rib heads contacting the aorta has not been described previously.

## Case report

Written informed consent to publish this case report was obtained from the patient.

A 36-year-old man was admitted to the emergency department of our level I trauma center after he was run over by a car at approximately 40 km per hour. On arrival, his level of consciousness was fluctuating, and his vital signs were unstable. He was complaining of chest pain and difficulty breathing and had a respiratory rate of 32 breaths per minute with an oxygen saturation of 88%. Physical examination revealed a lip laceration, a road rash covering 40% of his anterior abdominal surface, a large hematoma on the right flank, and pelvic tenderness. Portable radiographs of the chest and pelvis revealed multiple bilateral displaced rib fractures, a left clavicular fracture, small bilateral pneumothoraces, subcutaneous emphysema, and left lung patchy opacities concerning for pulmonary contusions, as well as multiple pelvic fractures with a left sacroiliac joint diastasis. Bilateral chest tubes were placed, and the patient was intubated. The focused assessment with sonography in trauma (FAST) was positive for intra-abdominal free fluid. The patient was taken to the operating room for an emergency exploratory laparotomy. He was found to have a large retroperitoneal zone 3 hematoma and a grade 3 splenic injury. A splenectomy and preperitoneal packing were performed. He also underwent angioembolization of bilateral internal iliac arteries. Post-operatively, he was transferred to the intensive care unit.

**Fig. 1:**
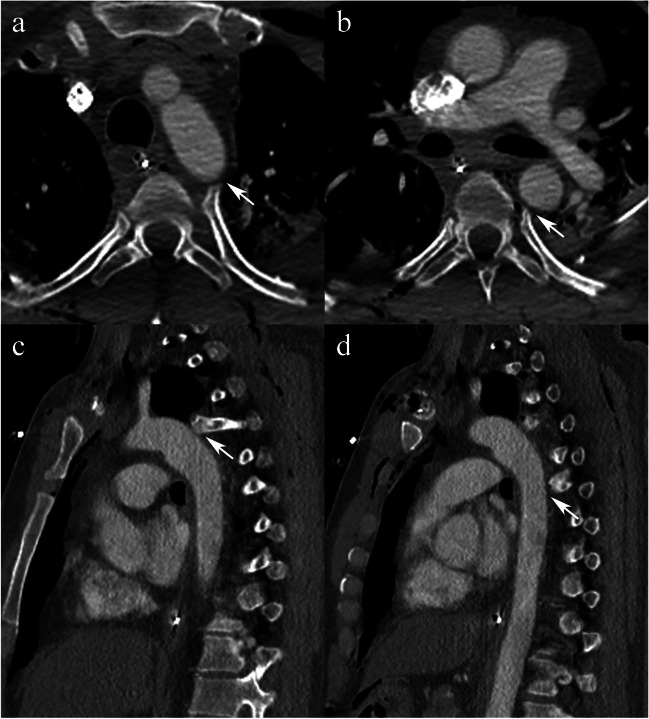
Initial post-trauma CT in 36-year-old male who was run over by a car, with axial (**a**) and sagittal (**c**) images demonstrating a left fourth costovertebral joint dislocation, and axial (**b**) and sagittal (**d**) images showing a left sixth costovertebral joint subluxation, with the heads of the ribs abutting the aorta (arrows)

A CT of the head, facial bones, cervical spine, thorax, abdomen, and pelvis obtained later the same day revealed multiple injuries, including bilateral hemopneumothoraces, pneumomediastinum, pulmonary contusions, grade 3 splenic injury, left adrenal hematoma, retroperitoneal hematoma, Morel-Lavallée lesions at bilateral hips, right acromioclavicular joint separation, left sacroiliac joint diastasis, and multiple fractures, namely, at the nasal bones, scapulae, clavicles, ribs, pelvis, right distal radius, and right L1 and bilateral C7 and L5 transverse processes. Regarding the thoracic cage injuries, on the right, there were fractures at the neck and at the costochondral junction of the first rib, and comminuted fractures of the second through seventh ribs posteriorly. On the left, there were fractures of the head and neck of the first rib; costal cartilages of the first, second, and fourth ribs; costochondral junctions of the fifth and sixth ribs; and body of the second through ninth ribs at more than one site except for the eight rib (flail chest). There was subluxation of the left fourth costotransverse joint and a fracture/dislocation of the left fourth costocentral joint; there was a tiny intra-articular chip fracture of the posterior aspect of the head of the rib. There was also a subluxation of the left sixth costocentral joint. The heads of both the left fourth and sixth ribs abutted the posterior aspect of the proximal descending aorta (Fig. 1). These costovertebral joint injuries and their proximity to the aorta were not described on the initial CT report.

The patient underwent multiple subsequent chest CTs for ongoing fever. The last scan performed 54 days post trauma demonstrated persistent costovertebral joints malalignment and no aortic injury. A transverse fracture through the superior endplate of T4 with no significant loss of height or involvement of the posterior wall was occult on the initial CT and became apparent on subsequent CTs.

Over the course of his hospitalization, the patient underwent surgical fixation of the pelvic fractures. His recovery was complicated by infarctions at the left posterior inferior cerebellar artery and right posterior cerebral artery territories. The patient was discharged to a rehabilitation center after a 75-day hospitalization. As the only subsequent follow-up in our electronic medical records, the patient visited the emergency department for an upper respiratory tract infection approximately 1 year after the trauma.

## Discussion

**Fig. 2:**
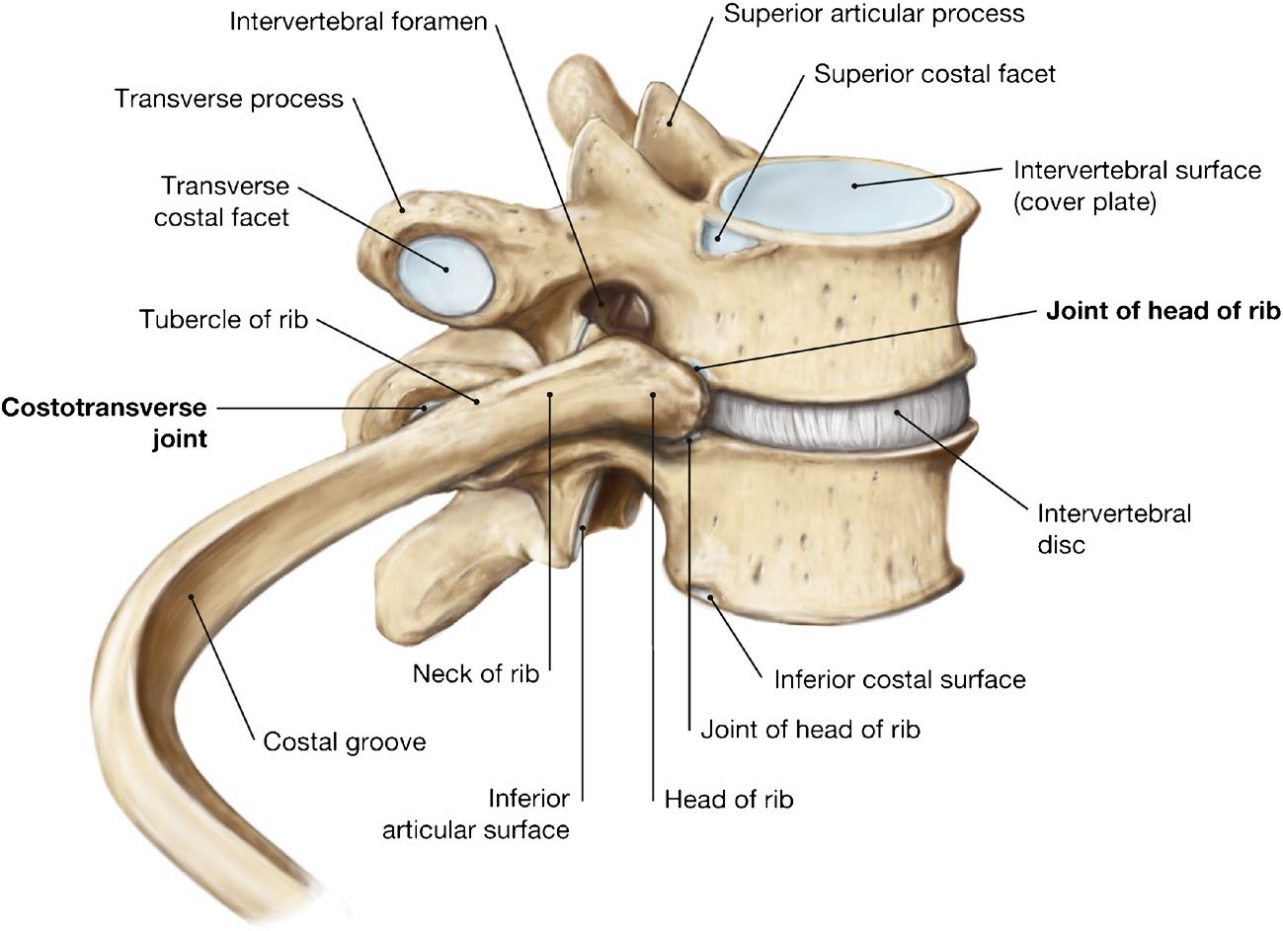
Illustration of the costovertebral joint anatomy, including the costotransverse joint and the costocentral joint (also known as joint of head of rib), at the level of the T7 and T8 vertebrae. Reprinted with permission from Hombach-Klonisch S, Klonisch T, Peeler J. Thorax. In: Hombach-Klonisch SK, Thomas., Peeler J, editors. Sobotta Clinical Atlas of Human Anatomy. 1 ed. Germany: Elsevier; 2019. p. 223-80, Figure 5.21

The rib cage typically comprises the sternum, twelve pairs of ribs and costal cartilages, and twelve thoracic vertebrae [39]. Anteriorly, ribs one to seven (i.e., true ribs) directly connect with the sternum via their costal cartilage, whereas ribs eight to ten (i.e., false ribs) indirectly join the sternum through the costal cartilage of their superior rib. Ribs eleven and twelve (i.e., floating ribs) do not connect with the sternum. Posteriorly, the ribs articulate with the spine via the costovertebral joints, which consist of the costocentral and costotransverse joints (Fig. 2) [6].

The costocentral joint of first, tenth, eleventh, and twelfth rib consists of a simple synovial joint with the correspondingly numbered vertebra [40]. The costocentral joint of the second through ninth rib is complex, with the two articular facets on the rib head articulating with the facets on the margin of the corresponding vertebral body and the vertebral body immediately above it, respectively, in two synovial compartments, as well as with the crest on the rib head attaching to the intervertebral disc via the intra-articular ligament. The costocentral joint is stabilized by the capsular, radiate, and intra-articular ligaments, although the latter is absent from the first, tenth, eleventh, and twelfth CVJs.

The costotransverse joint is comprised of the articulation between the facet on the tubercle of the rib with the costal facet on the transverse process of its corresponding vertebra [40]. This articulation does not exist at the eleventh and twelfth ribs. The costotransverse joint is supported by the capsular, costotransverse, superior and lateral costotransverse, and accessory ligaments. The first rib does not have a superior costotransverse ligament.

The costotransverse joint is more susceptible to injury than the costocentral joint [6]. The location of the first rib and last ribs at the edges of the rib cage as well as the differences of their CVJ anatomy and anterior cartilaginous attachment may increase their risk of dislocation [2, 6, 9, 12]. In the literature, we found that dislocations of the first rib [3–11] or the eleventh and twelfth ribs [9, 12–15] were most common. Few cases of fourth [3, 9, 16, 17] or sixth [9, 18] CVJ dislocation have been reported, with none of them presenting the involvement of these levels together. Another unusual feature of our case is the involvement of non-consecutive levels, which has not been reported previously. In our review of the literature, CVJ disclocations involved either a single level [4, 6, 8–12, 15, 16] or multiple consecutive levels [2, 3, 5, 7, 9, 13–15, 17, 18].

The thoracic cage plays an important role in stabilizing the thoracic spine by restricting motion and increasing stiffness [41, 42]. The addition of the sternal-rib complex as a fourth column has been suggested to complement the three-column model of the Denis classification system for thoracic spinal stability [43]. Injury to the costovertebral joints could compromise the spine’s capacity to bear physiological loads [42, 44]. As CVJ dislocations are often managed conservatively [2, 6–8, 10–14, 17, 18] and can remain misaligned, it would be interesting to study the long-term structural and biomechanical impact of CVJ instability on the thoracic spine, albeit such a study may be challenging giving the rarity of these injuries.

CVJ dislocations occur predominantly in young adults, and affect males [2–16, 18, 19] substantially more frequently than females [9, 17], which is consistent with the demographic characteristics of the 36-year-old male in our case report. Such injuries usually result from major trauma [6, 8, 15]. As our patient experienced, motor vehicle accidents are the most frequently reported cause of CVJ dislocation [2–7, 9, 10, 12–17]. Other reported causes include fall from height [8, 9, 18, 19], crushing trauma [9, 11], gunshot wound [9, 15], and helicopter blade injury [15]. It is believed that CVJ dislocations are primarily due to indirect forces, although direct forces could also potentially produce these injuries [12].

CVJ dislocations are frequently accompanied by other injuries. Rib fractures are a common finding in CVJ dislocations [2, 3, 5, 8, 9, 11, 13–18], while fractures of the clavicle [5, 6, 9, 11, 14], scapula [4, 11, 15], or sternum [2, 9, 15] may also occasionally be present. CVJ dislocations are strongly associated with spine fractures or dislocations [2–4, 6, 9, 13–15, 17–19] and can occasionally be accompanied by spinal cord injuries [9, 15–19]. In our case, a T4 vertebral body fracture was occult on the initial CT and became apparent on follow-up CTs. This highlights that, when CVJ dislocations are present, radiologists should carefully examine the spine for possible injuries. Intra-thoracic injuries, such as hemothorax [2, 3, 5, 11, 14, 17], pneumothorax [2, 5, 6, 14], lung contusion [2, 6, 14, 15], cardiac contusion [16, 18], and mediastinal hematoma [4, 16]; intra-abdominal injuries like splenic [6, 18], hepatic [15, 16], and renal [15] lacerations; and pelvic and extremity fractures [6, 10, 16] as well as brachial plexus injuries [5, 11] may also sometimes be seen in the setting of CVJ dislocations. CVJ dislocations have also been reported to be associated with mortality [9, 15], although they are never the cause of death. This finding likely reflects the severity of traumas in which CVJ dislocations are encountered. This also raises the possibility that, while CVJ dislocations may be rarely documented because their actual occurrence is rare, another explanation may be that they are rarely imaged because patients with these injuries in major traumas may not survive to hospital.

Rib fractures, particularly those involving the posterior left ribs 4–11 (most commonly 6–8) with anterior displacement, can injure the thoracic aorta [45]. Such injuries are life-threatening, and early diagnosis and treatment are critical for survival [26, 32]. Hemorrhagic shock resulting from traumatic aortic injuries may occur with a delay ranging from several hours to several days after initial trauma [25–36]. The reason for the late aortic rupture is not always clear [26, 28, 30, 34]. In some cases, it is possible that the aortic injury happened at the time of the initial trauma, but that hemostasis was achieved by a thrombus or tamponade effect from the fracture rib, which was later disrupted [26, 27]. In other cases, the sharp edge of a fractured rib could have resulted in continuous irritation leading to perforation or in a sudden new traumatic incident [25, 28, 31, 33]. The latter mechanism was suspected in reported cases where hemorrhagic shock occurred immediately after patient rolling and chest physiotherapy [25] or shoulder reduction [31]. Thus, caution with patient manipulation and positioning, a high index of suspicion for aortic injury in the setting of a new hemothorax with hemodynamic instability, and early surgical intervention for rib fracture fragments that pose a risk for aortic injury have been suggested [46].

Vascular injury from a left twelfth CVJ fracture-dislocation resulting in a subcostohemiazygos fistula has been described [47]. A disruption of the subclavian artery related to a right first CVJ dislocation in the absence of a fracture has also been reported [4]. Among the few reports of CVJ dislocation, most cases were managed conservatively [2, 6–8, 10–14, 17, 18], although surgery has been used in persistently symptomatic cases [9, 38]. To the best of our knowledge, the approach to CVJ dislocations where the displaced rib head contacts the aorta has not been described in the literature. Our patient was managed conservatively with no short-term complications, including no development of aortic injury on the follow-up CT performed 54 days after the trauma. Therefore, it is possible that the blunt rib head does not threaten the integrity of the aorta as would a sharp fracture fragment, supporting conservative management. However, long-term imaging follow-up data would be helpful to draw stronger conclusions on outcomes and potential complications associated with conservative management. It remains nevertheless critical for radiologists to describe the relationship of the dislocated rib to adjacent structures, to inform the treating physicians of potential risks, such as impingement on nerves or compression of vessels. Delayed thoracic aortic injuries have the potential to be missed on initial imaging [48]. In our case, the CVJ dislocations and the abutment of the rib head on the aorta were not mentioned in the initial report. In a study by Hsu et al., a sharp rib edge penetrating the aorta was missed on initial CT, contributing to a lethal delayed aortic rupture [27]. In a study by O’Brien et al., CVJ fracture-dislocations were missed prospectively on 63% of radiographs and 20% of CTs on which they were present [15]. This highlights the need for radiologists to be aware about these types of injuries to ensure that they are identified and reported.

In conclusion, this case report presented dislocations of non-consecutive left fourth and sixth CVJs where the rib head abutted the aorta. It documents an unusual presentation of a rare entity and suggests that conservative management may be acceptable in some of these cases. It also raises awareness among radiologists about these injuries, which can be easily overlooked, but are important for the stability of the thoracic spine. Finally, this report emphasizes the association between CVJ dislocations and spine injuries, which can sometimes be occult on initial imaging. Future studies with larger sample sizes and long-term follow-up could help determine the optimal management of CVJ dislocations.
